# P04.67. Survey on hand gestures relevance in patient practitioner communication: a homeopathic example

**DOI:** 10.1186/1472-6882-12-S1-P337

**Published:** 2012-06-12

**Authors:** M Escher, A Büssing, F Escher, T Ostermann

**Affiliations:** 1 University of Witten/Herdecke, Academic Practice, Hagen, Germany; 2 University of Witten/Herdecke, Center for Integrative Medicine, Herdecke, Germany

## Purpose

A systematic review showed that patients convey important informations in spontaneous co-speech hand gestures (HG) when presenting their complaints and describing their pain experiences. Only a minority of practitioners and therapists were reported to have actively analyzed patients’ hand gestures during case taking. Sensation method (SM) homeopaths were one of the reported exceptions. This survey was designed to gain a better understanding of the perspective, usage, appraisal and general relevance of manual co-speech gestures by SM homeopaths.

## Methods

Ninety-four out of 306 seminar attendants (mean age 49.6 y, 80.9% female, 57.4 % physicians and 42.6% healing practitioners) at two seminars on SM homeopathy with varying degrees of expertise, answered a standardized 54-item questionnaire. For 20 items on “perspective, utilization and relevance of gestures in patients’ symptom description” a factor analysis was performed, by means of principal components analysis and varimax rotation to arrive at a solution that demonstrates both the best simple structure and the most coherence. Nine items were excluded after reliability analysis due to low item total correlations.

## Results

Eleven remaining items formed a set of three factors explaining 66.6 % of variance. The first factor with five items describes “Hand gestures in relation to verbal expressions” (α = 0.81). The second factor includes four items regarding “Hand Gestures describing the experience of bodily and mental symptoms” (α = 0.74). The third factor is regarding the “practitioners’ behaviour and active attitude in observing hand gestures” (α = 0.86).

## Conclusion

This survey shows how SM homeopaths actively observe HG and judge them to help patients in expressing their symptoms qualities and illness experience. Whether this view is shared by other physicians or medical professions should be investigated in the future.

